# Supplementary material for: Spontaneous persistent activity and inactivity in vivo reveals differential cortico-entorhinal functional connectivity
Source: Nat Commun. 2024 May 8;15:3542. doi: 10.1038/s41467-024-47617-6 (PMC11079062; doi:10.1038/s41467-024-47617-6)
Supplement: Supplementary file 1 — Supplementary Information [file 41467_2024_47617_MOESM1_ESM.pdf]

Supplementary Information for Choudhary et. al.

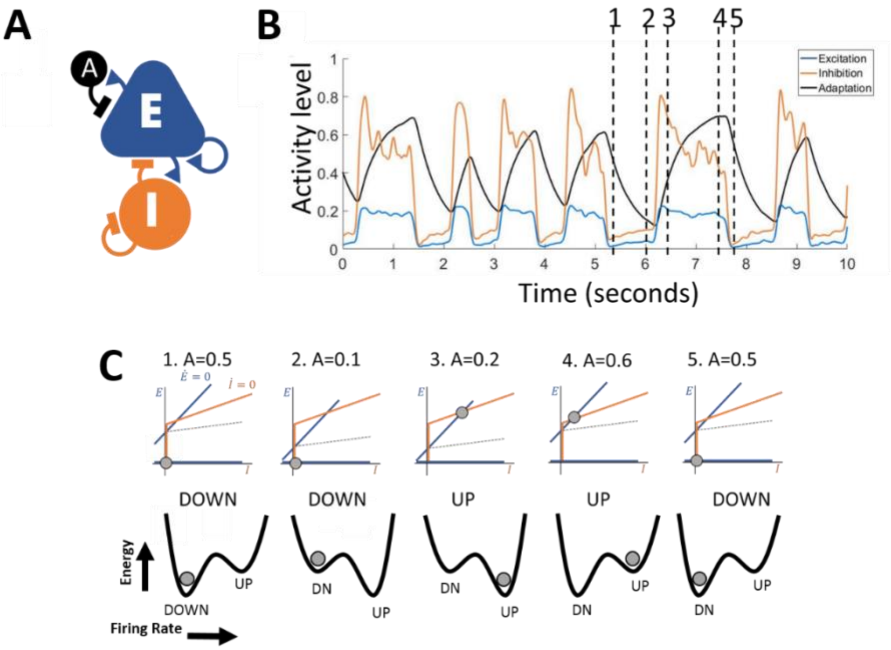

Supplementary Fig. 1: A single network of inhibitory and adaptation mediated excitatory populations can produce Up-Down state oscillations. **A)** Excitatory and inhibitory populations are recurrently connected, and their activities are quantified by variables *E* and *I*, which vary between 0 and 1 and represent the proportion of active (spiking) neurons in the population. Only the excitatory population has activity dependent adaptation α, which evolves with time constant τ_{α}=500s. **B)** A sample trace of *E, I,* and α. **C)** Top row: The time-evolution of the network for one complete UDS cycle (time points in B) can be visualized on the *E-I* phase space. Nullclines for the *E* and *I* variables are plotted in blue and orange, respectively, and denote where the time derivative for that particular variable ($\dot{E}, \dot{I}$) is zero. Intersections of the nullclines denote equilibrium points where both $\dot{E} = 0$ and $\dot{I} = 0$. There are two stable points, corresponding to the Up state (*E,I*>0) and the Down state (*E,I*=0). These two attractor points form basins of attraction in the *E-I* plane, with a separatrix (dashed line) denoting the boundary. Under noise, the relative stability of each point is inversely proportional to its distance from this separatrix, and the entire system can be viewed as an energy landscape, with each stable point as an energy minimum (bottom row). An increase in α corresponds to an upwards translation of the $\dot{E}$ nullcline, making the Up state less stable; a decrease in α corresponds to a downward shift, making the Down state less stable.

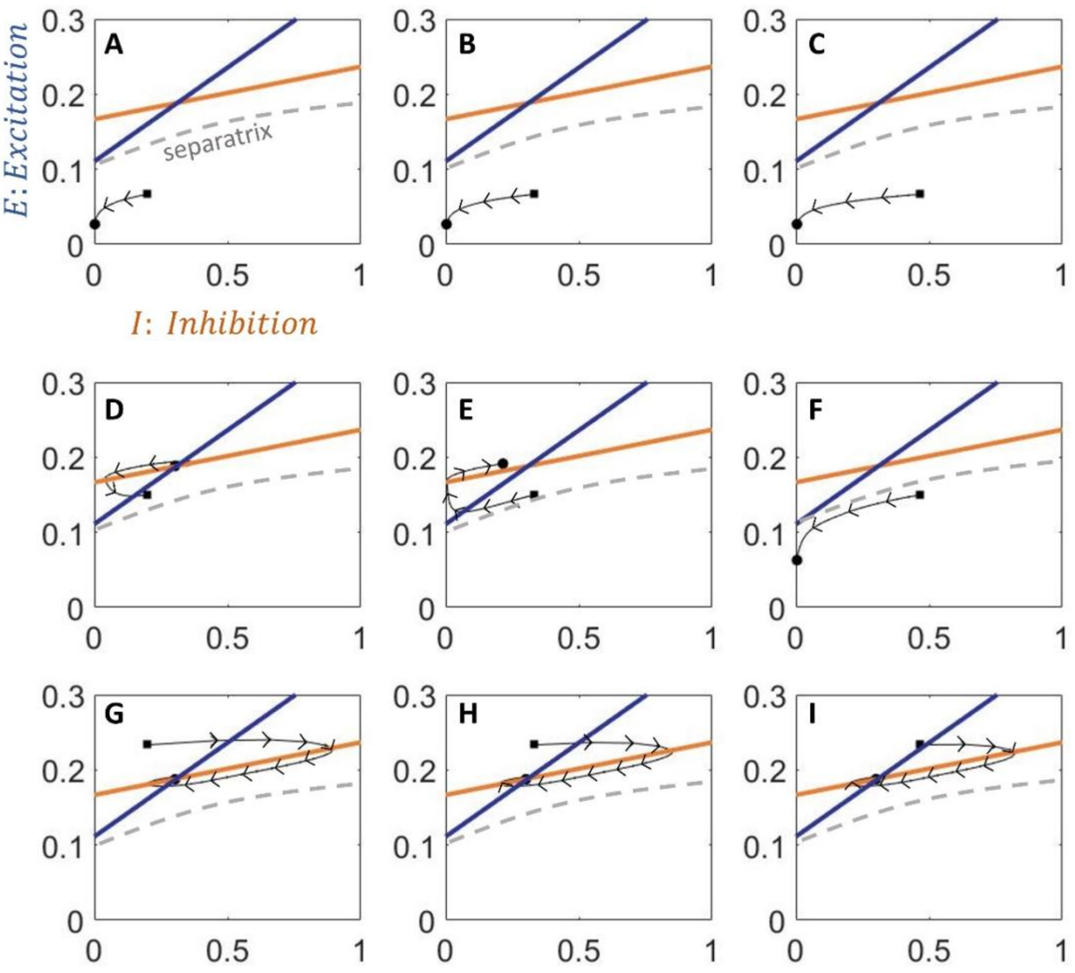

Supplementary Fig. 2: The mean field equations define an attractor landscape in the *E-I* coordinate plane. The mean field model defines two discrete attractors in the *E-I* coordinate plane. Here we trace the evolution of a single network in the (E, I) coordinate plane from various initial conditions (denoted by the black square) to the final condition (denoted by the circle) in the absence of noise and for fixed adaptation level (α=0.5). The excitation and inhibition null clines are depicted (solid colored lines) along with the separatrix (dotted line). Conditions A, B, C, and F end in the 'Down' state fixed point, while the others (D, E, G, H, I) end in the 'Up' state fixed point.

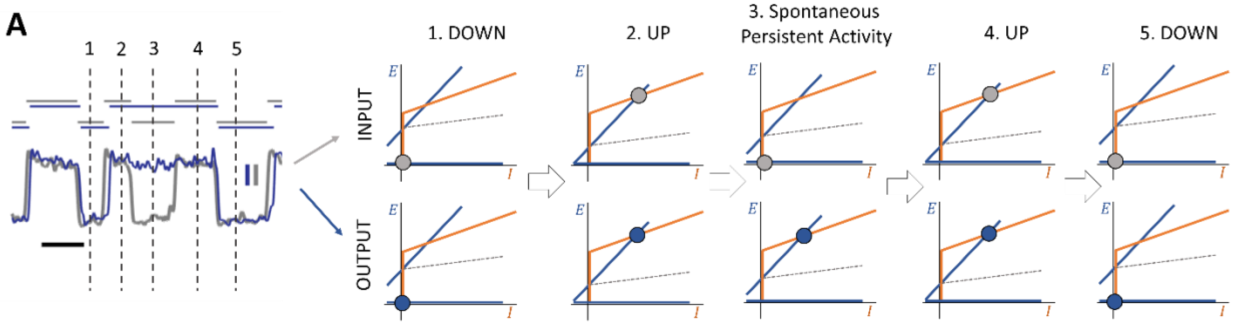

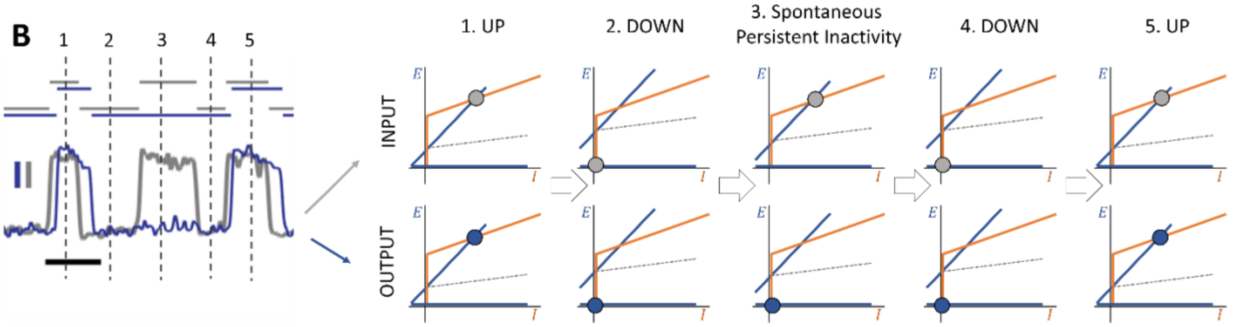

Supplementary Fig. 3: *SPA* and *SPI* occur when the efferent network persists in its current state and does not follow a state transition in the afferent network. A) Left: *SPA* occurs when the efferent network (blue) persists in the Up state while the afferent (gray) undergoes a complete Down state. The black scale bar represents 1 sec in time, and the blue and gray scale bars correspond to activity of 0.1. Right: Each network lives in its own *E*-*I* coordinate plane. A sudden decrease (from 2->3) in the afferent input (an Up-Down transition) translates the efferent output *E* nullcline upward, destabilizing the Up state and inducing a synchronous transition to the Down state. If the destabilization is not enough (because the decrease was not large, i.e. the afferent Down state has high activity), the efferent network can persist in the Up state on its own, resulting in *SPA*. **B)** Similar to A, but showing *SPI*, which occurs when the efferent network persists in the Down state while the afferent undergoes a complete Up state. A sudden increase in the afferent input (a Down-Up transition) translates the efferent *E* nullcline downwards, destabilizing the Down state an inducing a transition to the Up state. Again, if the destabilization is not enough (because the afferent Up state has low activity), the efferent network can persist in the Down state, resulting in *SPI*. Scale bars show time (horizontal black, 1 second) and the amplitude in afferent (vertical gray, 0.1) and efferent (vertical blue, 0.1) network activity.

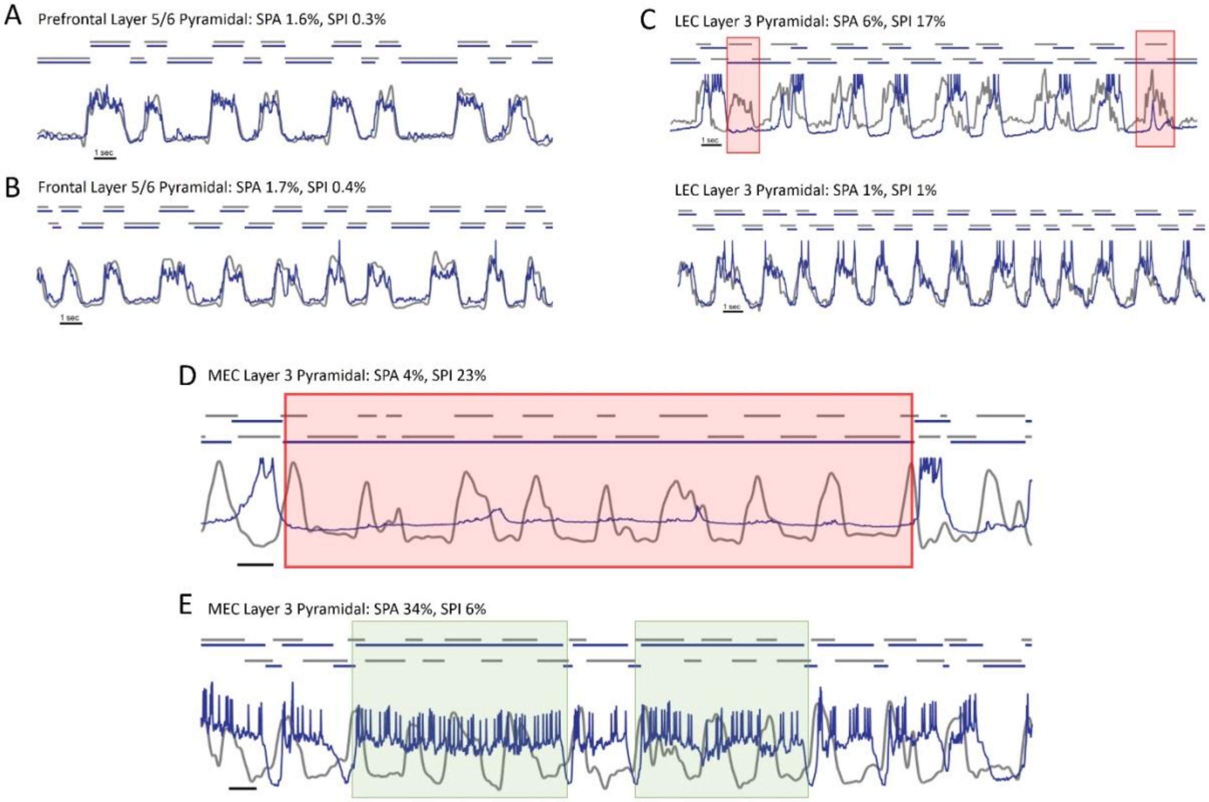

Supplementary Fig. 4: Additional examples of experimental traces A) Example trace of the V_m (blue) of a prefrontal cortex layer 5/6 pyramidal neuron, along with simultaneously recorded neocortical LFP (gray). Black scale bar at the bottom shows one second time interval, and the detected Up-Down state sequence is shown above for each trace. **B)** Example trace from frontal cortex layer 5/6 pyramidal neuron. Both neurons (in A and B) exhibited phase-locked UDS to the neocortical LFP. **C)** Two examples of LECIII pyramidal neurons, one showing heightened levels of persistent inactivity (top, red boxes), and the other showing complete phase locking (bottom). **D)** An example of MECIII neuron showing extremely long persistent inactivity state (red box), lasting over 17.2 seconds. **E)** An example of MECIII cell showing extremely long persistent activity states (green boxes), lasting 8 and 6 seconds.

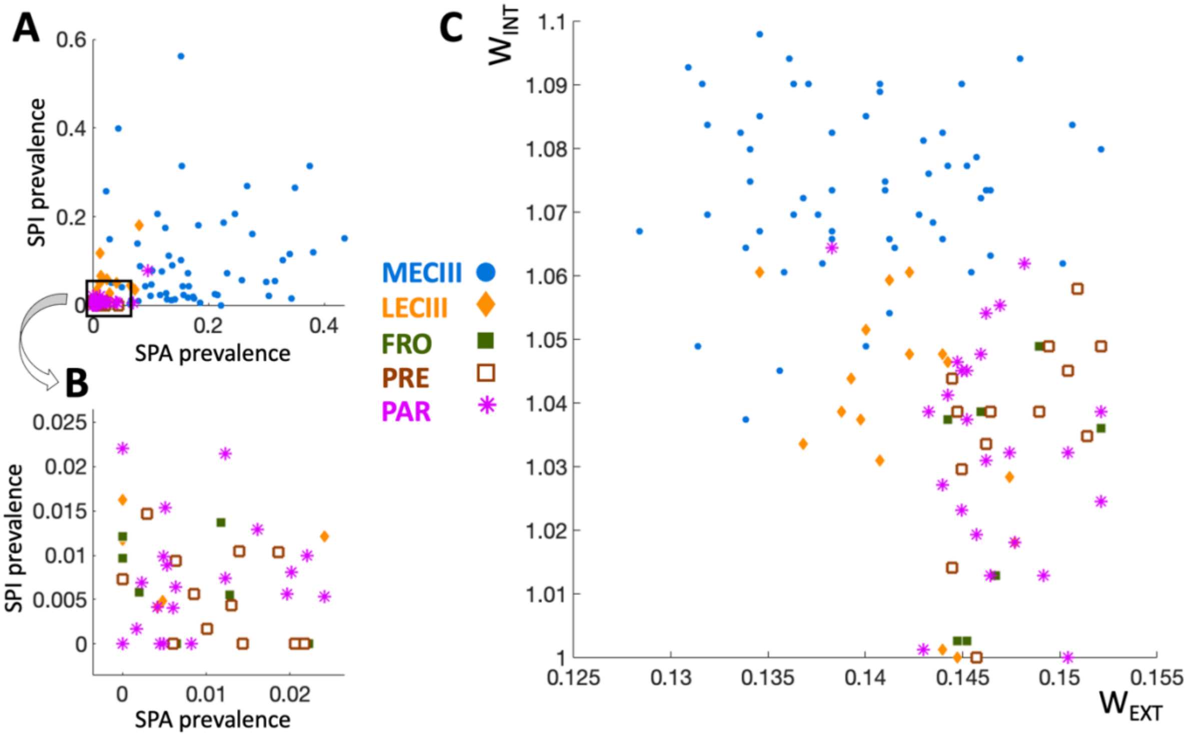

Supplementary Fig. 5: The amount of *SPA* in a n entorhinal cell was not significantly correlated with the amount of *SPI*., but Similarly, fitted parameters W_{INT} and W_{EXT} are not significantly anti-correlated. A) Both MECIII and LECIII Ccells showed varied levels of *SPA* and *SPI* in experiment, but there was no significant correlation between the average *SPA* and *SPI* exhibited by a cell. (MECIII: r=0.18, $p>10^{-1}$; LECIII: r=0.49, $p>10^{-1}$). **B)** A close up of the lower box in (A) shows distribution of neocortical cells. These also shows no statistically significant relationship (Spearman's rank correlation). **C)** The fitted parameters for each cell were also not significantly correlated with one another (Spearman's rank correlation).

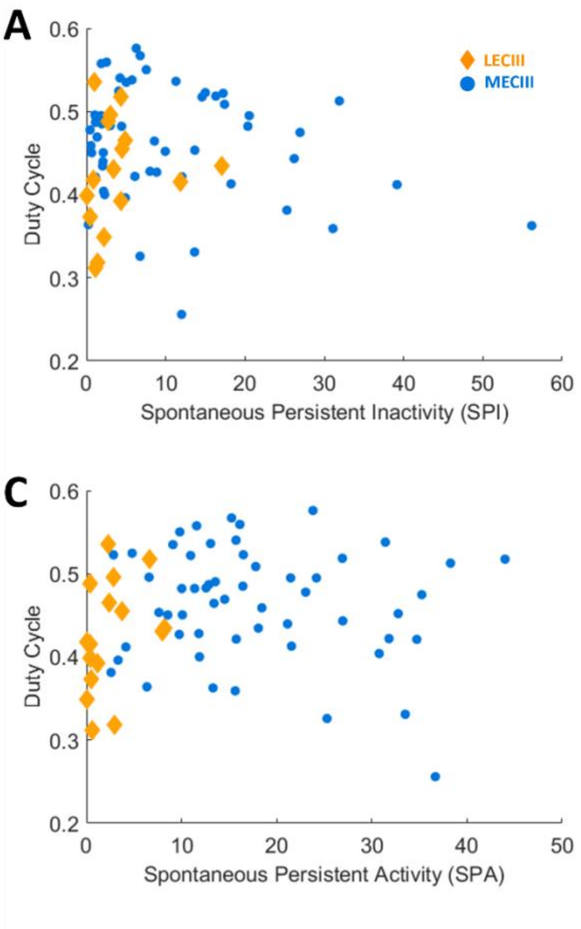

Supplementary Fig. 6: The prevalence of *SPA* or *SPI* in an experiment was independent of the depth of anesthesia. The depth of anesthesia varies from one experiment to another, and deeper anesthesia produces UDS with longer duration cycles and smaller duty cycle (proportion of the cycle spent in the Up state). Neocortical UDS statistics were used to quantify depth of anesthesia of the animal. **A)** *SPI* prevalence was not significantly correlated with neocortical duty cycle for both LECIII neurons (yellow: r=0.15, p>0.1) and MECIII neurons (blue: r=-0.24, p>0.05). **B)** *SPI* was not significantly correlated with mean duration of neocortical UDS cycles in the experiment (LECIII: r=-0.27, p>0.3; MECIII: r=0.17, p>0.2). **C)** Conversely, *SPA* was not significantly correlated with neocortical UDS duty cycle (LECIII: r=0.38, p>0.3; MECIII: r=-0.12, p>0.3) and **D)** not correlated with mean duration of UDS (LECIII: r=-0.43, p>0.05; MECIII: r=-0.01, p>0.9). Statistical test used: Spearman's rank correlation.

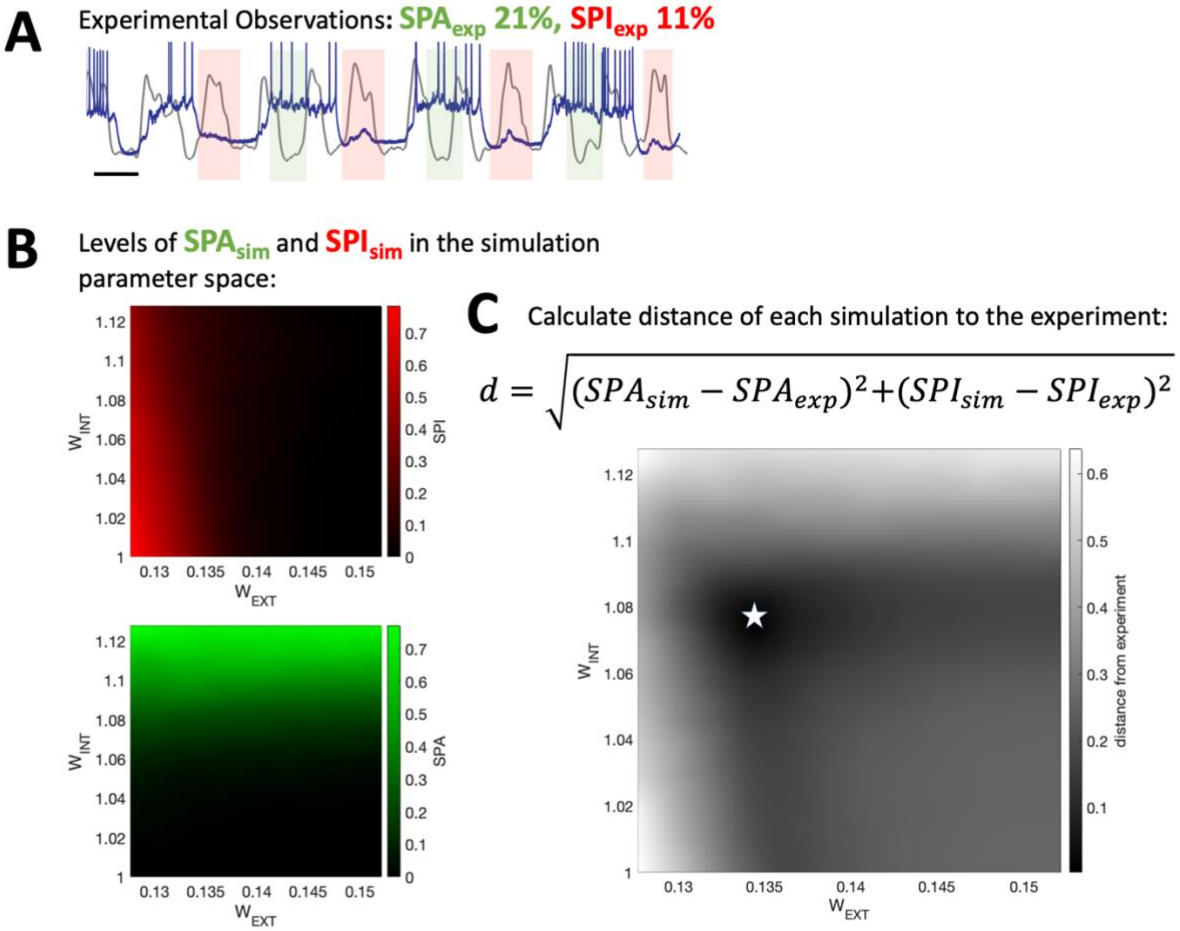

Supplementary Fig. 7: Fitting procedure matches observables SPA and SPI to infer W_{INT} and W_{EXT}. A) Example of simultaneous recording of parietal LFP (gray) and MEC III membrane potential (blue), with observed level of persistent activity and inactivity in the experiment. **B)** The level of SPA (below, green) and SPI (red, above) as observed in the simulation space. **C)** To fit the experiment observed in (A) to the simulation, we minimize a Euclidean distance metric between each simulation and the experiment. The coordinates of the minimum (white star) represents the inferred connectivity parameters W_{INT} and W_{EXT}.

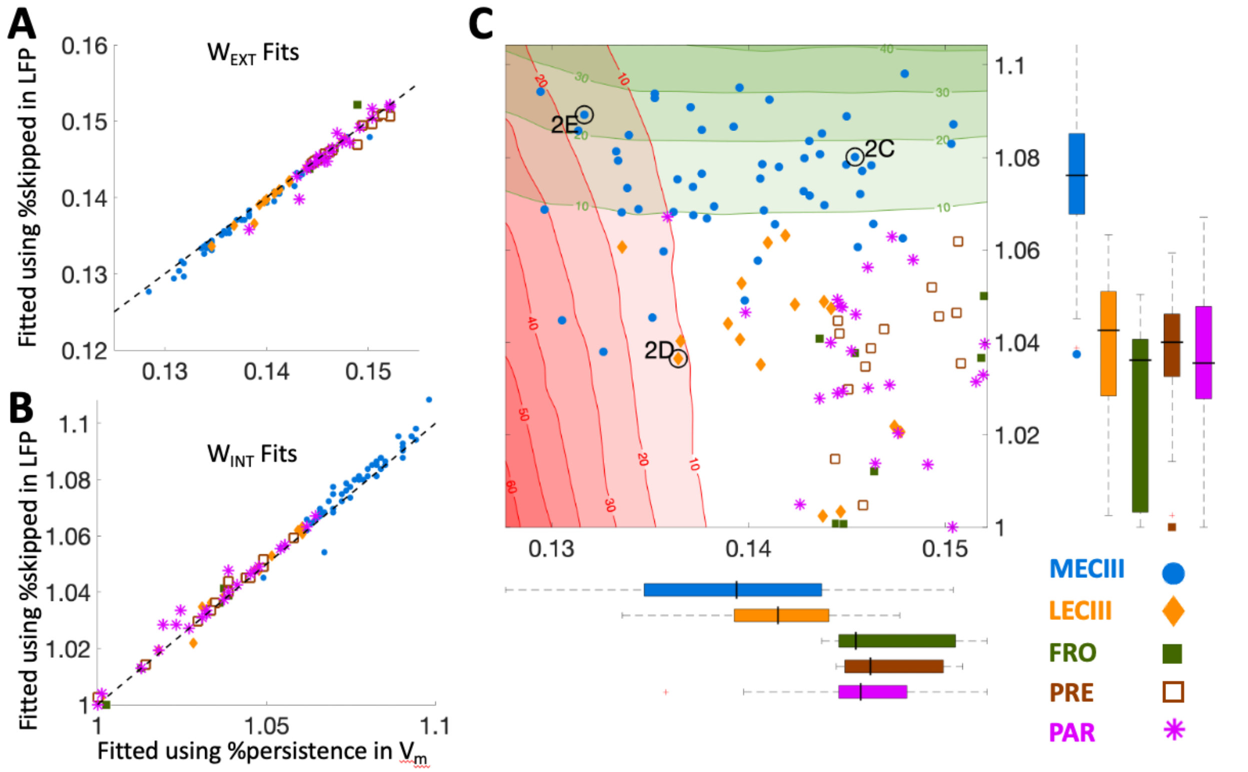

Supplementary Fig. 8: Experimental fits to the model were robust over different fitting procedures. In the main text (see Fig 3b), the time-averaged prevalence of *SPA* and *SPI* in the efferent states was used to match simulations to the experiment. Another method of fitting experimental data to the model is to use the time averaged proportion of *afferent* states that are "skipped" by the efferent network (skipping afferent Up states leads to *SPI*, while skipping afferent Down states leads to *SPA*). Each cell is matched to the simulation space using this metric. The symbols used for each brain region are the same as in Fig 2A. **A)** Comparing the fits to W_{EXT} resulting from this matching scheme (x-axis) to the %*SPA*/*SPI* scheme used in the main text (y-axis) reveals that both methods give extremely close values (diagonal line is perfect agreement). **C)** Comparing the fits to W_{INT} also shows stark agreement. **C)** The parameter space similar to Fig 1,3 in the main text, but the colors and contour lines show % afferent states skipped instead of % efferent SPA/SPI. The results for connectivity and the relationships between regions is preserved.

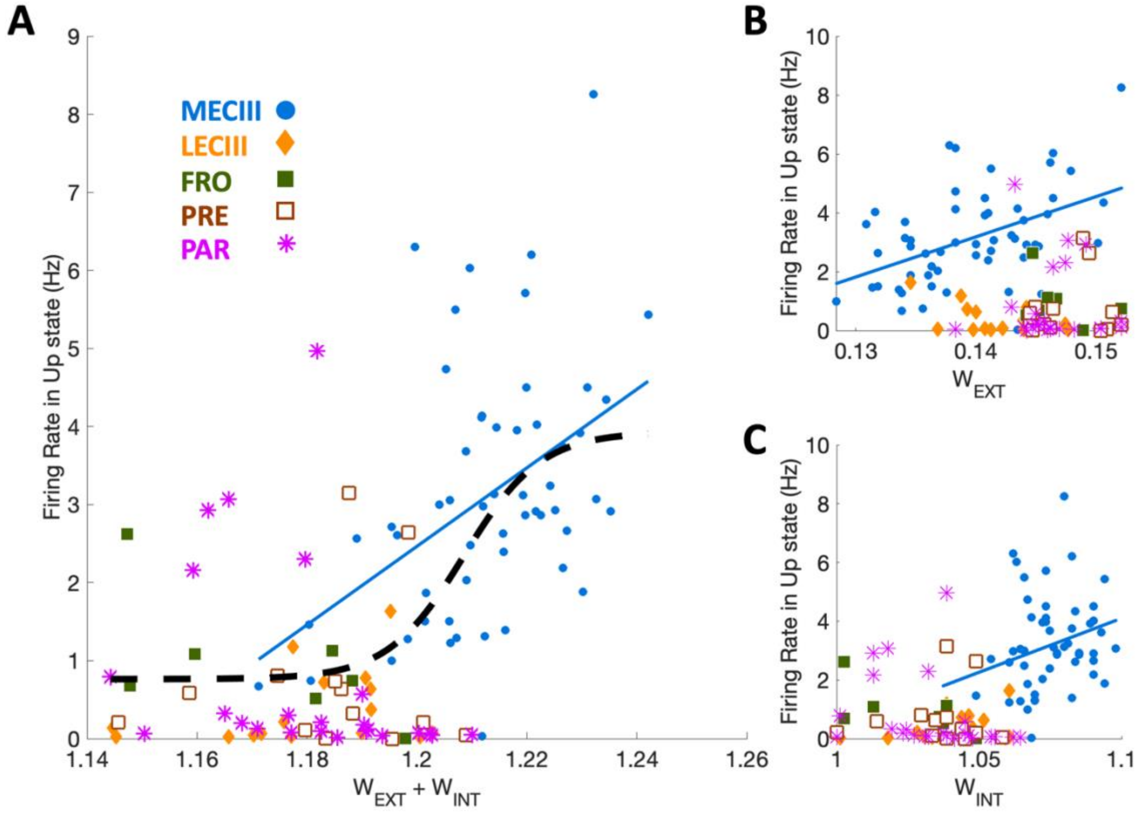

Supplementary Fig. 9: Firing rate of MECIII but not LECIII cellscells was significantly correlated to inferred connectivity parameters. A) To find the total excitatory input into a neuron, we used the sum of our estimates for the recurrent and external excitations $W_{EXT} + W_{INT}$. The MECIII population showed significant positive correlation (blue line, r=0.49, $p<10^{-3}$) while the LECIII population did not show significant correlation (r=-0.29, $p>10^{-1}$). When all cells are treated equally, the data is consistent with a nonlinear function (black dotted line), consistent with theories that show nonlinear relationship between input and output in pyramidal neurons. **B)** The average firing rate in the Up state was significantly correlated with our estimate for the external input W_{EXT} for only the MECIII population (r=0.43, $p<10^{-3}$) and not for the LECIII population (r=-0.4, $p>10^{-1}$). **C)** The average firing rate of a neuron during the Up state was not significantly correlated with our estimate for the recurrent excitation W_{INT} for either the MECIII (blue: r=0.07, p>0.5) or LECIII (yellow: r=0.42, $p>10^{-1}$) populations. All correlations were calculated using Spearman's rank correlation coefficient.

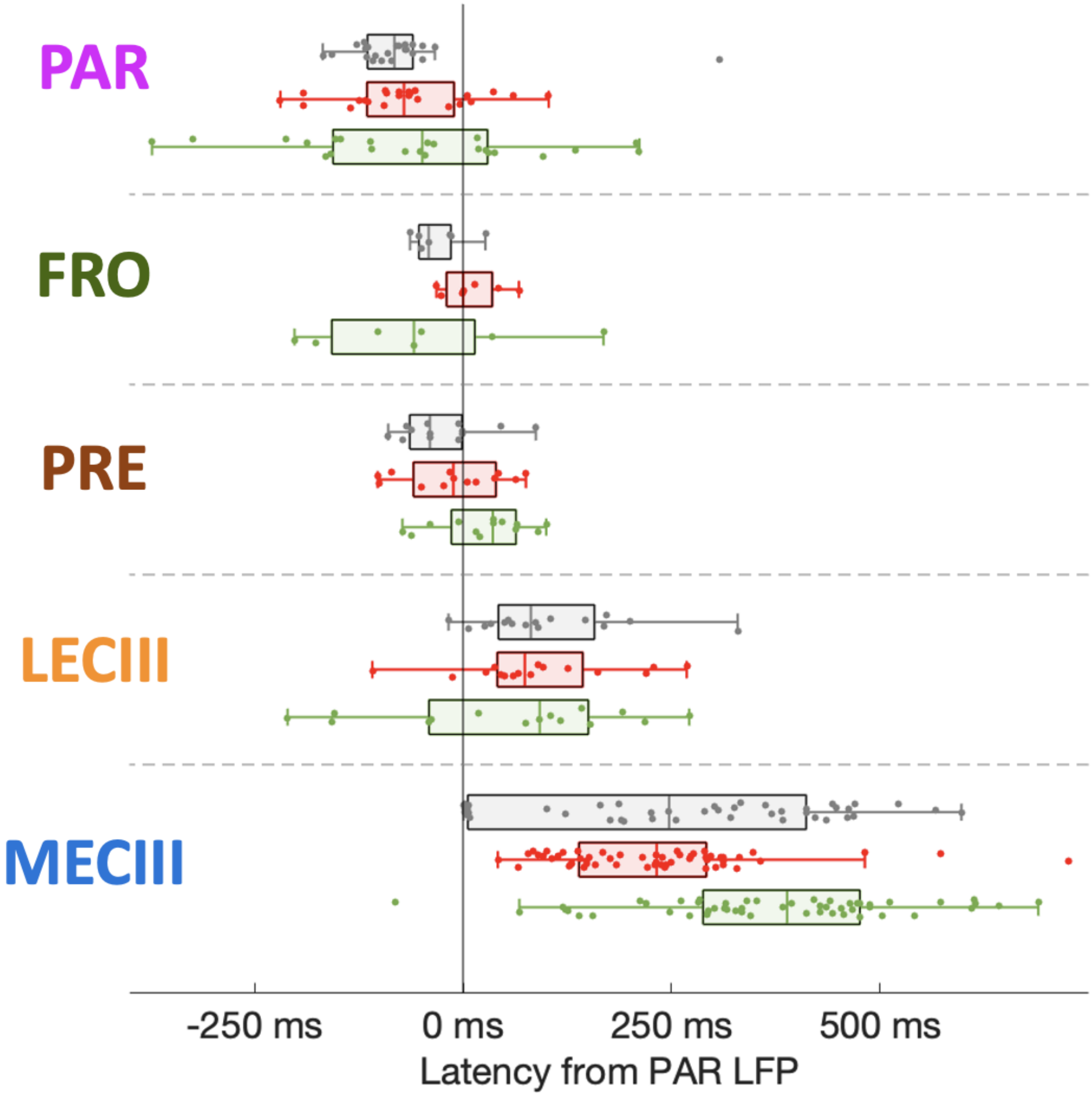

Supplementary Fig. 10: Up to Down transition lag between afferent neocortical LFP and ECIII V_m is most correlated with recurrent excitation W_{INT}, while the Down to Up transition lag is most correlated with external input strength W_{EXT}. Same as main text Fig 4a. The internal recurrent excitation W_{INT} increases the stability of efferent network Up state, leading to higher Up-Down delays w.r.t. neocortical LFP. Box edges indicate the 25th and 75th percentile, and center black bar denotes the median. The numerical values can be found in the Sup. Table 1.

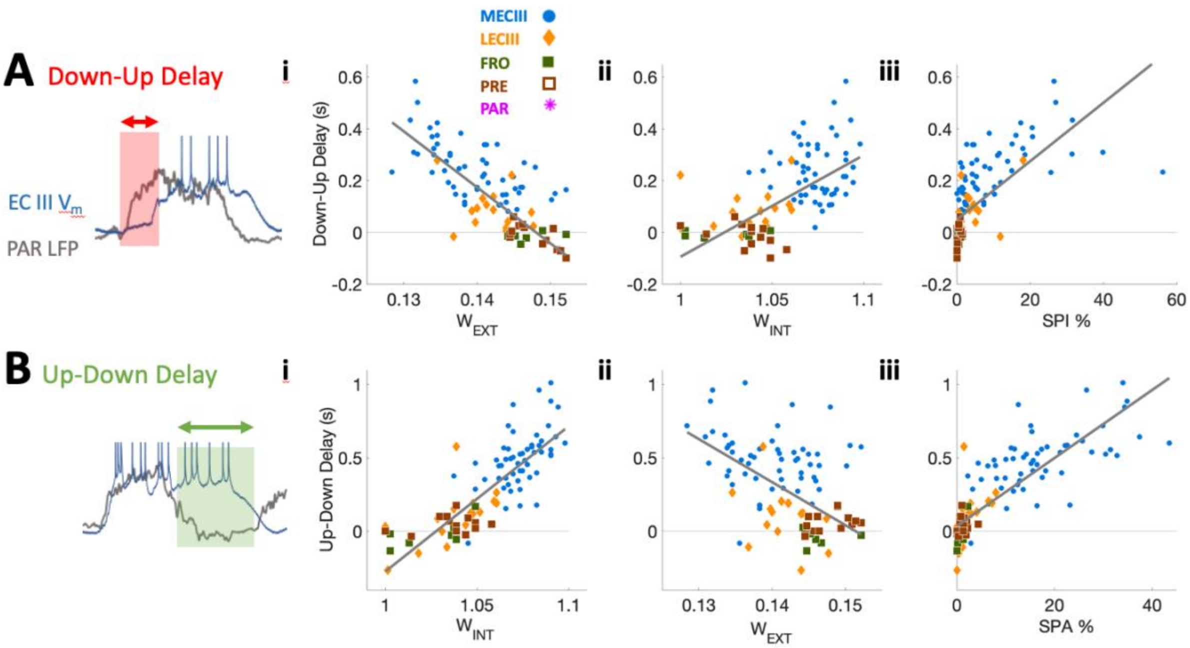

Supplementary Fig. 11: Up to Down transition lag between afferent neocortical LFP and ECIII V_m is most correlated with recurrent excitation W_{INT}, while the Down to Up transition lag is most correlated with external input strength W_{EXT}. Ai) Same as main text Fig 4d/e. The internal/external recurrent excitation $W_{EXT/INT}$ increases/decreases the stability of efferent network Down/Up state, leading to longer/shorter Down-Up/Up-Down delays w.r.t. neocortical LFP (MECIII: blue, $r=0.47$, $p<10^{-3}$; LECIII: yellow, $r=-0.663$, $p<10^{-162}$). All delays are reported in units of mean UDS duration. **Aii)** The Down-Up delay is positive correlated with the internal W_{INT} connectivity ($r=0.545$, $p<10_{-10}$). **Aiii)** The amount of *SPI* in a neuron was correlated with the Down-Up delay ($r=0.571$, $p<10^{-11}$), but this correlation was less significant compared to our model's findings for connectivity in Ai. **Bi)** Same as main text Fig 4G. W_{INT} is positively correlated with Up-Down delay, as the internal excitation stabilizes the Up state ($r=0.823$, $p<10^{-30}$). **Bii)** W_{EXT} is negatively correlated with Up-Down delay, as a larger W_{EXT} would mean that more input would be cut from the efferent network, making the shutdown of activity sooner ($r=-0.53$, $p<^{-10}$). **Biii)** Previous studies reported a strong correlation between the amount of *SPA* in a neuron and the Up-Down delay. This is reproduced here ($r=0.820$, $p<10^{-30}$) and is not significantly different to the correlations found by our model. Same as Fig 4g. W_{EXT} is significantly correlated with the Down to Up Delay (MECIII: $r=-0.56$, $p<10^{-5}$; LECIII: yellow, $r=-0.60$, $p<10^{-2}$). All correlations were calculated using Spearman's rank correlation coefficient.

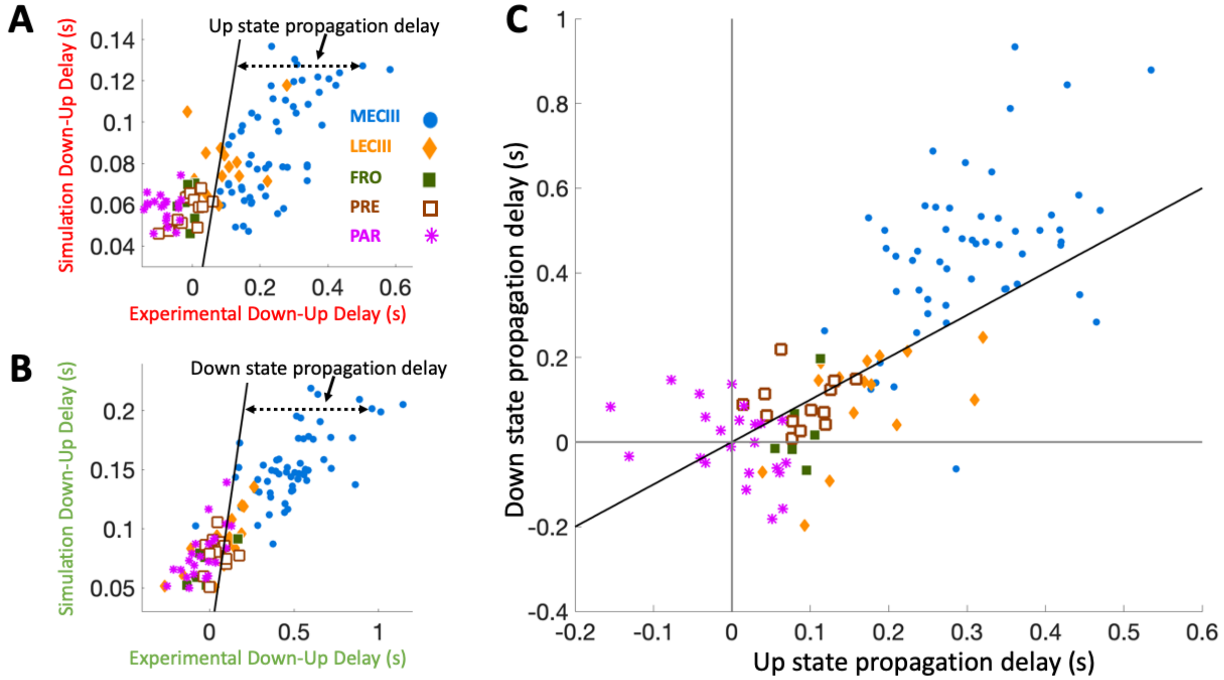

Supplementary Fig. 12: Model can make quantitative predictions about latency, which are highly correlated with the experimentally observed values. A) The experimentally observed Down-Up delay (x-axis) of each neuron was different from the predicted Down-Up delay (y-axis), given the fit of W_{INT} and W_{EXT} calculated from levels of SPA and SPI. The black diagonal line everywhere shows the y=x line, where both would be equal. The difference between the experimental value and the predicted value was taken as the Up-state signal propagation delay, which was calculated separately for each neuron. **B)** Similarly, the Down-Up delay of each simulation differed from the experimentally observed value. The difference was taken as the Down-state signal propagation delay. **C)** When comparing the signal delay between the Down state and the Up state, the two are correlated across cells. The MECIII cells show significantly higher Down state propagation delay vs. Up state propagation delay ($p<10^{-3}$, two-sided nonparametric Wilcoxon rank-sum test).

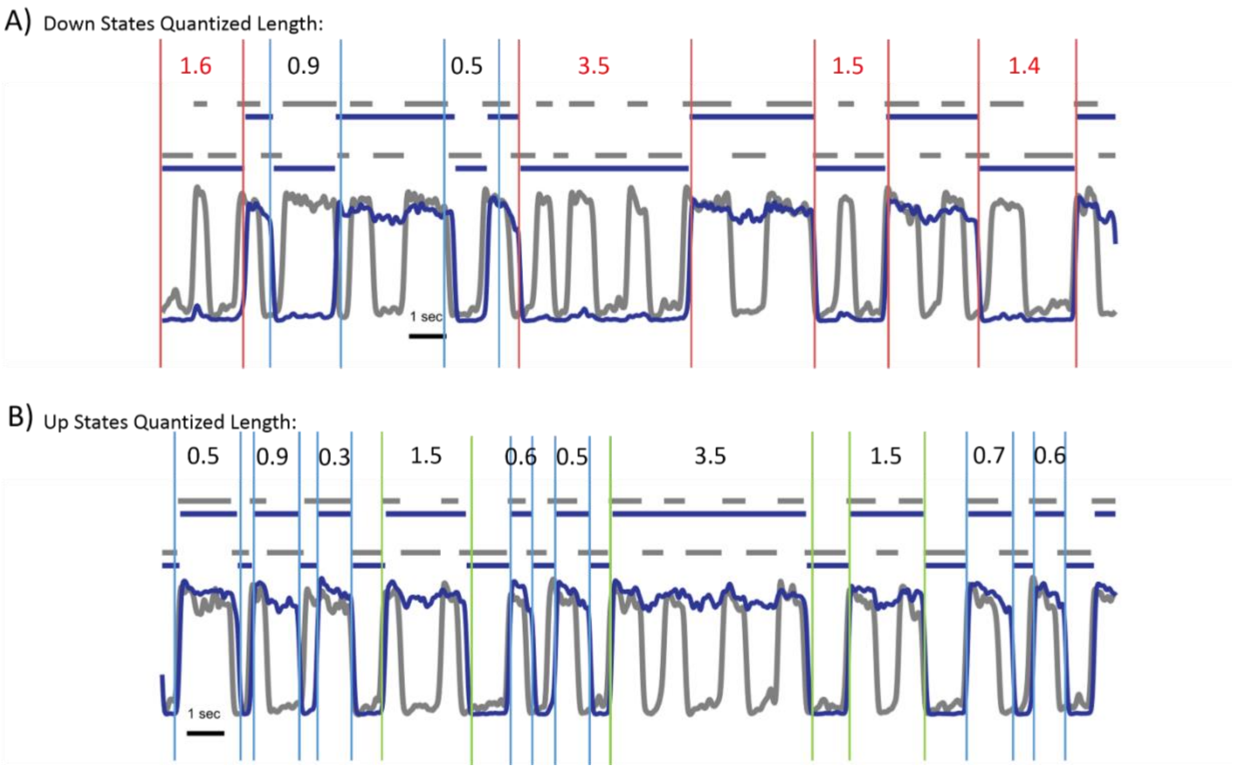

Supplementary Fig. 13: Counting membrane potential state durations in terms of neocortical UDS cycles. A) 'Down' states in the membrane potential (blue trace) are first aligned with the nearest neocortical 'Down' state, and the number of LFP 'Down' and 'Up' states this particular membrane potential 'Down' state lasts is counted. Each 'Down' and 'Up' state receives a time length of 0.5 units, a full UDS cycle is thus 1-unit long. The 'Down' states in red represent those that are considered "spontaneous persistent inactivity," since they last for longer than one full UDS cycle. **B)** Same as A, but for 'Up' states. Those 'Up' states that were identified as persistent activity are highlighted in green.

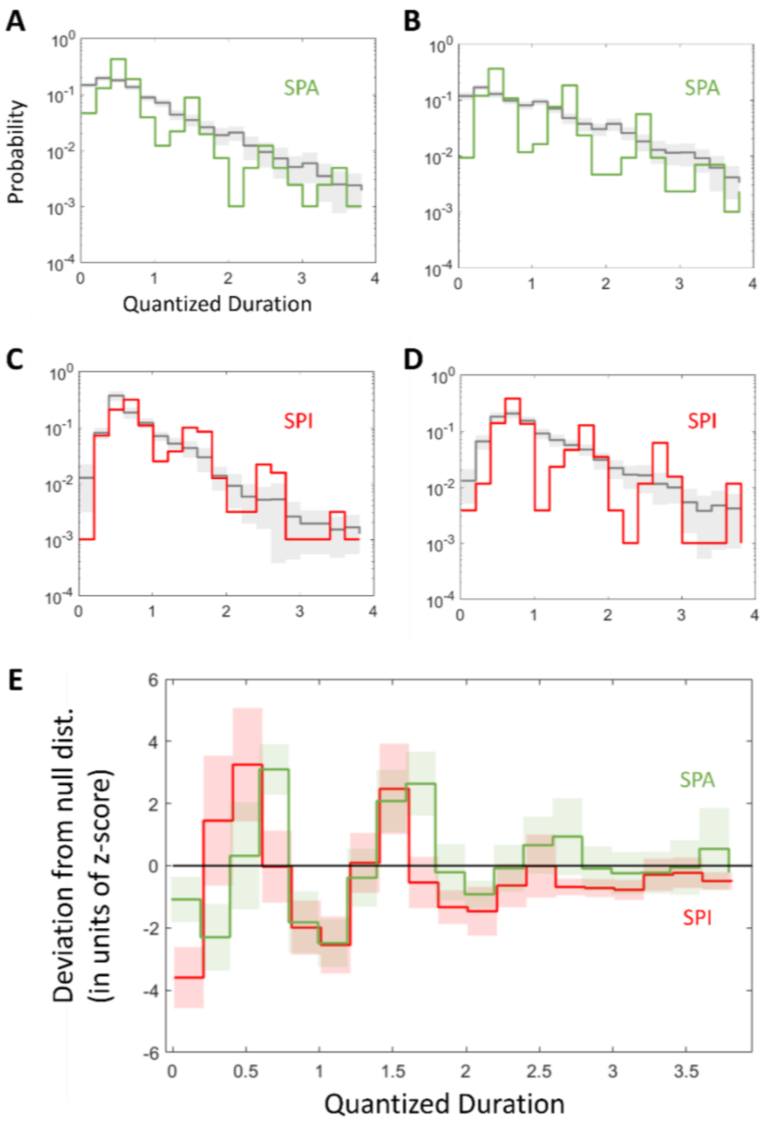

Supplementary Fig. 14: Experimental distributions of efferent state length in units of afferent UDS was significantly quantized compared to bootstrap shifted null distributions. A) The distribution of efferent Up state lengths in terms of afferent UDS in the experiment (green) was compared with the same distribution resulting from a random shift of the efferent data (gray). The standard deviation (gray shaded area) of the null distribution was obtained by calculating the distribution from 60 random shifts. The peaks and troughs of the real distribution were significantly different from the null. **B)** Another example of efferent *SPA* quantization, with corresponding null distribution. **C)** An example of efferent Down state lengths (red) in terms of afferent UDS also shows significant quantization, different from the null distribution (gray). **D)** Another example of significant quantization of *SPI*. **E)** Taking the difference between the real and null distributions from each experiment and normalizing by the standard deviation in each bin shows that the first trough (at quantized duration (QD) =1) and the second peak (at QD=1.5) for both *SPA* and *SPI* is significantly different (*SPA*: first trough $p<10^{-3}$, second peak $p<10^{-3}$; *SPI*: first trough $p<10^{-3}$, second peak $p<10^{-2}$) from the null distribution at the population level. The SPA QD peaks were shifted to the right of the SPI peaks, but the troughs occurred at the same QD. This reflects the fact that for MECIII neurons the Up-Down latency is significantly larger than the Down-Up latency, and that Up state termination is internally determined, while Up state initiation is affected strongly by external input.

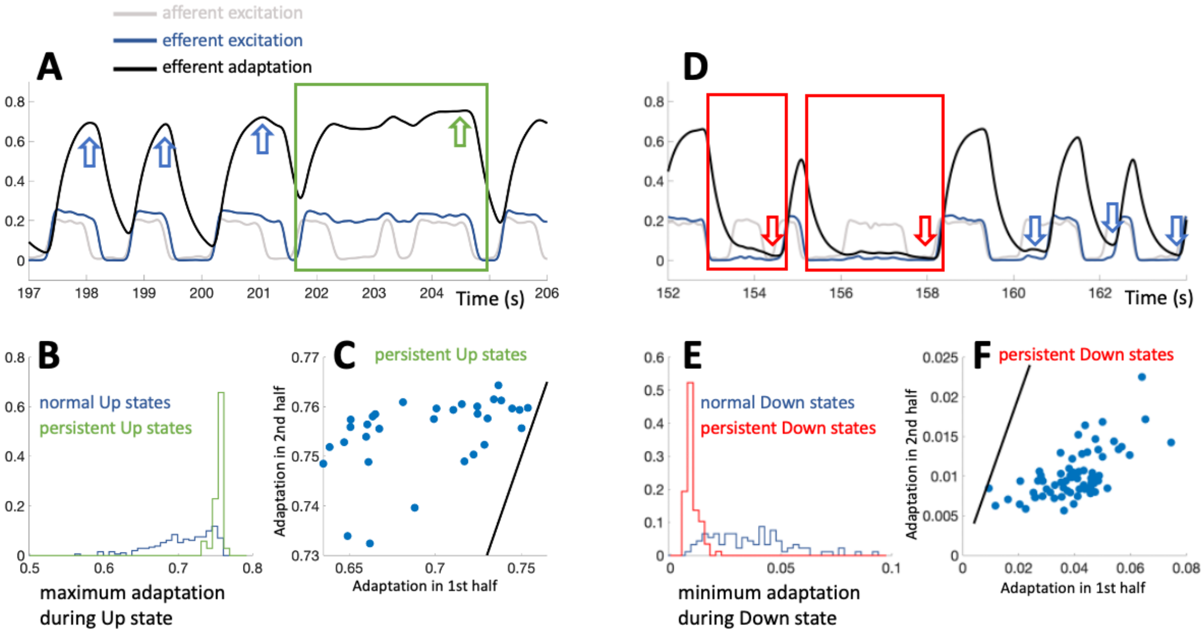

Supplementary Fig. 15: History dependence of SPA/SPI stems from the saturation of adaptation during persistent states. A) A trace from the model, showing afferent excitation (gray), efferent excitation (blue), and efferent adaptation (black). The green boxes highlight instances of persistent activity in the efferent network. Note that the efferent adaptation remains high during a skipped Down state and climbs even higher during the subsequent joint Up-state (green arrow) when compared to a synchronous Up state (blue arrows). **B)** The maximum value of efferent adaptation achieved during normal, simultaneous Up states (blue) was significantly lower than during persistent Up states (green), showing adaptation saturated at its highest level during persistent Up states. **C)** During persistent Up states, the adaptation during the second half of each Up state was always higher than during the first half (black line shows equality). Higher adaptation increases the probability of a Up-Down transition (see dynamical systems analysis), and thus the efferent network is more likely to go to the Down state after persistent activity. As a result, **D)** Same as A, but for a network showing SPI in red boxes. Note the efferent adaptation remains low during a skipped Up-state and falls even lower during the subsequent joint Down state (red arrows). **E)** The minimum value of efferent adaptation achieved during normal, simultaneous Down states (blue) was significantly higher than during persistent Down states (red), showing adaptation saturated at its lowest level during persistent Down states. **F)** During persistent Down states, the adaptation during the second half of each Down state was always lower that during the first half (black line shows equality). Lower adaptation increases the probability of a Down-Up transition (see dynamical systems analysis), and thus the efferent network is more likely to go to the Up state after persistent inactivity.

Region	UTD (ms)	DTU (ms)	Xcorr Lag (ms)
PAR	-49 ± 147	-71 ± 79	-82 ± 88
FRO	-58 ± 127	0 ± 35	-41 ± 31
PRE	35 ± 55	-11 ± 60	-40± 50
LEC III	92 ± 146	74 ± 95	81 ± 87
MEC IIII	389 ± 288	232 ± 123	247 ± 418

Supplementary Table 1: Values for latency between parietal LFP and V_m of neurons in each brain region. UTD: Up-to-Down delay. DTU: Down-to-Up delay. XCorr Lag: lag from cross correlation of the two time traces. All values are given in milliseconds.

Region	E cells (10^3) /mm^3	I cells (10^3) /mm^3	E:I ratio
Posterior Parietal Areas	68.7 ± 10.1	18.3 ± 3.6	3.75 : 1
Frontal Pole, Layer 2/3	84.9 ± 2.1	23.2 ± 0.6	3.65 : 1
LEC Layer 3	59.9 ± 4.8	15.6 ± 1.2	3.83 : 1
Dorsal MEC Layer 3	99.5 ± 12.6	15.3 ± 1.9	6.50 : 1
Ventral MEC Layer 3	207.7 ± 8.2	19.9 ± 0.7	10.4 : 1

Supplementary Table 2: Density of cell types in each brain region, as documented by EPFL's Blue Brain Cell Atlas initiative.
